# Metabolism of fatty acids and lipid hydroperoxides in human body monitoring with Fourier transform Infrared Spectroscopy

**DOI:** 10.1186/1476-511X-8-28

**Published:** 2009-07-24

**Authors:** Satoshi Yoshida, Qin-Zeng Zhang, Shu Sakuyama, Satoshi Matsushima

**Affiliations:** 1 Dept Biomol Sci, Fac Eng, Gifu Univ, Gifu, Japan; 2 Hebei Center for Disease Control and Prevention (CDC), PR China; 3 Cetr Res Lab, Mandom Co, Japan

## Abstract

**Background:**

The metabolism of dietary fatty acids in human has been measured so far using human blood cells and stable-isotope labeled fatty acids, however, no direct data was available for human peripheral tissues and other major organs. To realize the role of dietary fatty acids in human health and diseases, it would be eager to develop convenient and suitable method to monitor fatty acid metabolism in human.

**Results:**

We have developed the measurement system *in situ *for human lip surface lipids using the Fourier transform infrared spectroscopy (FTIR) – attenuated total reflection (ATR) detection system with special adaptor to monitor metabolic changes of lipids in human body. As human lip surface lipids may not be much affected by skin sebum constituents and may be affected directly by the lipid constituents of diet, we could detect changes of FTIR-ATR spectra, especially at 3005~3015 cm^-1^, of lip surface polyunsaturated fatty acids in a duration time-dependent manner after intake of the docosahexaenoic acid (DHA)-containing triglyceride diet. The ingested DHA appeared on the lip surface and was detected by FTIR-ATR directly and non-invasively. It was found that the metabolic rates of DHA for male volunteer subjects with age 60s were much lower than those with age 20s. Lipid hydroperoxides were found in lip lipids which were extracted from the lip surface using a mixture of ethanol/ethylpropionate/*iso*-octane solvents, and were the highest in the content just before noon. The changes of lipid hydroperoxides were detected also *in situ *with FTIR-ATR at 968 cm^-1^.

**Conclusion:**

The measurements of lip surface lipids with FTIR-ATR technique may advance the investigation of human lipid metabolism *in situ *non-invasively.

## Background

FTIR-ATR (Fourier-transform infrared spectroscopy with attenuated total reflection) technique is an established measurement system for analysis of chemical and biological materials and many applications have been developed. About 20 years ago this FTIR technique was applied to the microscopy for development of micro-spectroscopy [1] and imaging of chemical and biomedical materials, as this technique could be used for many kinds of materials in solid or liquid non-destructively. However, this FTIR measurement in millimeter scale, which may be especially important for analysis of macroscopic human body, has not been improved much so far. Although many important data have been accumulated concerning to the measurement of chemical and pathophysiological changes of human tissues and biomolecules with FTIR, the development of FTIR instrument for application to human body and tissues *in vivo *has not been advanced. In order to diagnose human body, especially the surface of skin or tissues non-invasively and easily, FTIR technique has a significant potential and it may be applied to detect important biomolecules, even though they are mixed in biological tissues and the depth profile in the skin may not be obtained. It could be used sophisticated statistical methods, such as partial least squares regression or other chemometrics [2,3] for analysis of components in complex mixtures. This FTIR method thus may be used, if it is available for all people, for chemical diagnosis of human body as such that the hand-held blood pressure measurement instrument was used for physical diagnosis of human vessels for all people at home.

FTIR-ATR technique may have several merits to measure changes of biomolecules on the surface of human body or tissues when comparing with laser Raman micro-spectroscopy. The latter laser Raman method may employ near-infrared laser and large hardware including photomultiplier, and the illumination of laser light on human skin, especially face skin, may be largely restricted. On the other hand, FTIR-ATR may be applicable to human body without risk. The application of FTIR-ATR to human skin (stratum corneum) was reported [4]. Recently a handy type of FTIR-ATR system was developed and this type would be applicable to investigation of human body easily if the target surface site to be measured could be correlated with the change of body conditions and analysis software was suitably used.

We focus now on the lipid metabolism of human body, and if we could develop FTIR-ATR system for measurement of lipid metabolism of human body non-invasively, this would greatly contribute to the advancement of management of people's health. However, the lipids on the usual skin surface may be mainly originated from sebum, and the sebum lipids may not reflect immediate changes of human body lipids. Normally the ingested lipids with foods were digested and adsorbed through intestine to blood and to liver, and resorbed through vessels to peripheral tissues, including skin tissues. It may be essential to find out sites of human body, especially skin tissues, where the lipid and fatty acid changes of blood were reflected nearly immediately, to measure non-invasively with FTIR-ATR.

We found, as shown in this paper, that the change of human lip surface lipid compositions reflected well that of ingested lipids, and the changes of lipids and fatty acids were really detected with FTIR-ATR *in situ*. This paper is the first to demonstrate that the measurement of lip surface lipids with FTIR-ATR non-invasively could show the actual metabolic rate of fatty acid from the intake of food to the appearance in the peripheral tissue.

## Methods

### Materials

Organic solvents were purchased from Aldrich-Sigma (USA), Kanto Chemical Co., and Wako (Japan). Lipid standards were purchased from Sigma(USA) and Funakoshi Co.(Japan). Silica gel thin layer plates were from Whatman(USA), and SepPak Silica cartridges were from Waters(USA). Durapore PVDF membrane filter (0.22 μm pore size) was from Millipore(USA). DMPD (N, N'-dimethyl-*p*-phenylenediammonium dichloride) was from Kanto Kagaku Co. (Japan).

### Analysis apparatus

**Figure 1:**
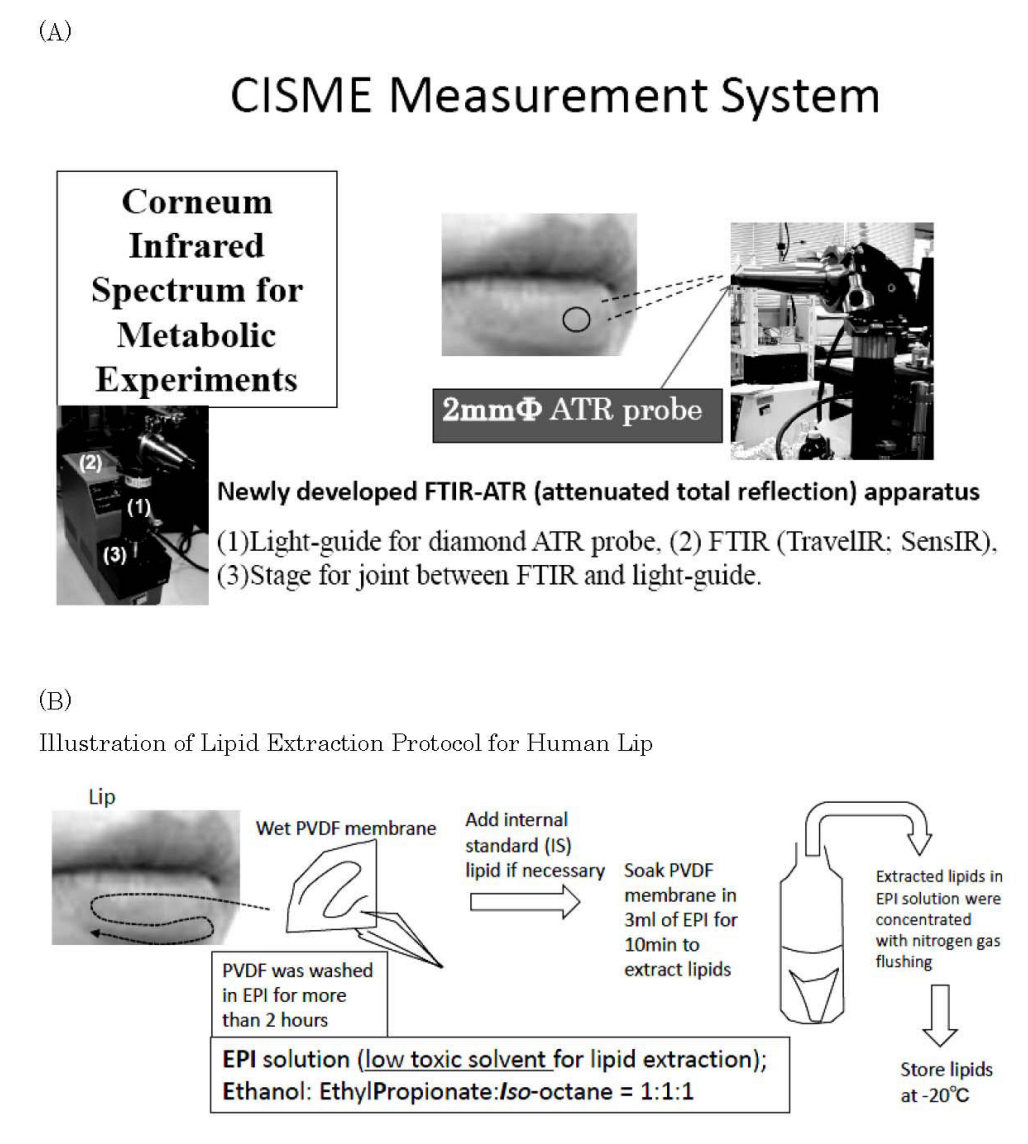
**(A). In situ measurement system for human lip surface with newly developed FTIR-ATR adaptor**. The probe diameter is 2 mm. Here we call this system CISME (Corneum Infrared Spectrum for Metabolic Experiments) measurement system. Fig. 1 (B). Illustration of extraction procedure for lip lipids using PVDF membrane and EPI solvents. Initially the washed lower lip was wiped at least 4 times with partially wetted PVDF membrane by EPI-solvent.

Fourier-transform infrared spectroscopy with attenuated total reflection adaptor (FTIR-ATR) (TravelIR, SensIR (now Smith Detection Inc.) with analysis software Grams-AI (Galactic Inc.). Specially designed ATR adaptor was made by the financial support of JST (Japan Science and Technology Agency, Tokyo, Japan) with S. T. Japan Co.(Tokyo, Japan) (shown in Fig. 1).

Fatty acids were analyzed with gas chromatography-mass spectrometry (GC-MS; GC-Mate II, JEOL, Japan) with capillary column (DB-WAX, 0.25 mm φ and 30 m long, GL Sciences Inc., Japan) after methyl esterification by 5% HCl-methanol at 100°C for 1 hr and extraction by hexane.

### Lipid Extraction Methods in Small Scale

#### Bligh/Dyer method (established standard method using toxic solvents)

Liquid sample in 1 vol (saliva, serum or blood; normally diluted to 0.5 mL with water) was mixed with 4 vol of a mixed solvent (chloroform:methanol = 1:2) vigorously, shaking for 1 min. Stand the mixture (homogenous solution) for 2 hrs at room temperature. Then add 1.3 vol of chloroform and 1.3 vol of water, and mix well, resulting in two layers. Centrifuge the mixture at 1500 g for 5 min, and two clear layers were obtained. The lower layer (chloroform layer) was collected by a Pasteur pipette after removing the upper layer, and concentrate the extracted lipids with flushing of nitrogen gas or evacuation.

#### EPI method (less toxic method developed in this paper)

Commercially available ethylpropionate should be distilled and purified at 40~50°C. Liquid samples such as saliva and blood are initially diluted to 0.5 mL (1 vol) by water, and **e**thanol in 3 vol (1.5 mL) and ethyl**p**ropionate in 1 vol (0.5 mL) and concentrated acetic acid in 1/50 vol (10 μL) were mixed well resulting in homogenous solution. Stand the mixture at room temperature for 2 hrs, and then further add a PI solvent (mixture of ethyl**p**ropionate:***i****so*-octane(1:1); PI) in 4 vol (2 mL) and mix with shaking for 1 min. Stand the mixture for 5 min. Then centrifuge the mixture at 1500 g for 10 min, resulting in two clear layers. Collect the upper layer for all lipids, and concentrate extracted lipids with flushing nitrogen gas.

### Staining of tape-stripped lip tissue

The adhesive tape for patch-test (round-shaped, Torii Pharmaceutical Co., Japan) was attached on lower lip vermilion portion, and the stripped surface tissue was stained with Alcian blue staining kit (sGAG Alcian Blue Binding Assay Kit, Funakoshi, Japan) to visualize the lip surface sulfated glycosaminoglycans. The stained tissue in blue was observed by a microscopy (Olympus, Japan).

### Detection and quantitative analyses of lipids by thin-layer chromatography (TLC)

**Figure 2:**
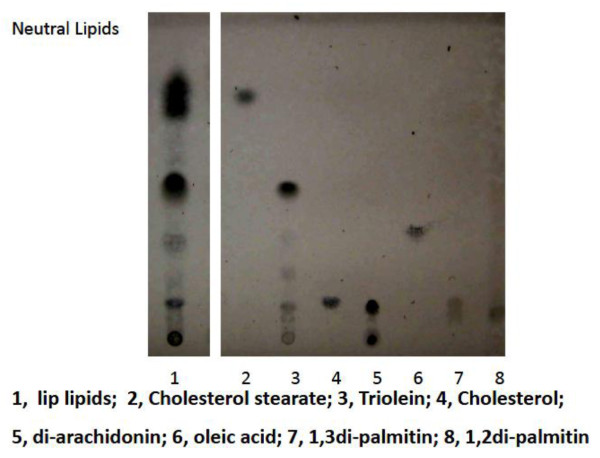
**Typical silica gel-thin layer chromatographic pattern of lip neutral lipids**. Developmental solvent used was hexane/diethylether/acetic acid (85:25:1). The extraction efficiency of lipids from PVDF membrane to EPI solvent was 50 ~60%.

Lipids were extracted from lower lip surface with wiping by PVDF membrane (shown in Fig. 2), which was initially washed for two hours and partially wet with EPI solvents (**E**thanol: Ethyl**P**ropionate: **I**so-octane = 1:1:1). The extracted lipids were re-dissolved in EPI solvent for 10 min, and dried by nitrogen gas flushing. The lipids re-dissolved in chloroform/methanol (1:2) solvent mixture were applied on silica-gel 60 plate for TLC analyses. Lipids on the TLC were normally visualized by 50% sulfuric acid spraying and charring at 110~130°C. Lipid hydroperoxides were visualized by DMPD (1% in methanol/water/acetic acid (128:25:1) mixture) spraying. Quantitative analysis of the neutral lipids of lip surface on TLC was carried out using the external standard (elaidic acid (C18:1Δ9t) methyl ester) and the stained band intensities were measured using a gel-plotting macro-programming software (Scion Image for Windows, Scion Co.).

### Silica- cartridge fractionation of neutral lipids

Silica-cartridges (Sep-Pak Plus, Waters Co., USA) were used for fractionations, and initially the cartridge was washed by 100% chloroform and then 100% methanol, and equilibrated with 100% methanol. The lipid sample in 100% methanol was applied on the cartridge with syringe, and the first elution as Fraction I was obtained with chloroform: methanol (95:5) mixture, and the second elution as Fraction II with chloroform: methanol (6:4), and the third elution as Fraction III with methanol (100%).

### Measurement of lip surface FTIR-ATR spectra for human volunteers in various ages

The FTIR-ATR measurement system (called CISME system here) for human lip was described in Fig. 1. The measurement of human volunteers with this system was approved from the Ethical Review Board of Gifu University School of Medicine (2007) and was in agreement with the Helsinki declaration. Initially the subject was informed the object and detailed procedure of the measurement, and after the informed consent of the subject was obtained, the lip of subject was measured with this FTIR-ATR system. The FTIR-ATR system was set on a desk and the subject was demanded to sit down on the chair in front of the CISME ATR probe. The lip (lower vermilion portion) of the subject after washing with facial soap and tap water more than six times and wiping the lip surface with paper towel was attached firmly on the diamond ATR probe and the measurement of FTIR was carried out for nearly 40 seconds with 8 cm^-1 ^resolution and 64 spectral accumulations. With this procedure dietary materials on the lips during feeding were almost washed off. Basically the diets for the volunteer subjects included the DHA yogurt contained 0.6 g DHA-triglyceride and 0.15 g EPA-triglyceride in 100 g plain yogurt (containing also ~0.05 g linoleic acid) which was taken during the breakfast. As the control diet, 0.75 g of soy bean oil in the 100 g yogurt was taken instead of DHA and EPA oils. Those DHA-yogurt and control-yogurt diets were fed in addition to the normal breakfast diets dependent on the individuals. For the age 60s volunteer subjects, the individual breakfast was taken at 6:30 a.m. ~7:30 a.m., and the lunch (the same non-oily Japanese-style 'udon' noodles in nearly 200 g for all subjects) was taken at 12:15 ~12:30. Here, *ex*., 12:30 means clock time twelve thirty, or 12 and a half, and so forth. For age 20s volunteer subjects, the breakfasts were taken between 7:00 a.m. and 9:30 a.m., and the measurements were carried out three times at 10:00 a.m., 11:00 a.m. and 12:00. In the normal diet, 100 g of cooked rice contained ~0.1 g linoleic acid as polyunsaturated fatty acids mainly, and ~50 g of cooked vegetables contained ~0.1 g of polyunsaturated fatty acids.

## Results

Fig. 1(A) shows FTIR-ATR system for measurement of lip (especially the labial vermilion zone) surface developed in our laboratory, namely CISME (**C**orneum **I**nfrared **S**pectrum for **M**etabolic **E**xperiments) measurement system. With this system, the diamond ATR probe with 2 mm diameter is pressed onto any point of lower lip surface, usually the center of lower lip, and the measurement was completed within 40 seconds.

Fig. 1(B) shows the procedure of lipid extraction from lip surface using EPI solvent (described in Methods section), and briefly the washed and slightly-wet PVDF membrane with EPI solvent was used to wipe lip surface quickly with pressing onto the lower lip, then the PVDF membrane with lip lipids was soaked in EPI solvent for 10 min to extract lipids, and the solvent was concentrated with nitrogen gas flushing and stored at -20°C until use.

**Figure 3:**
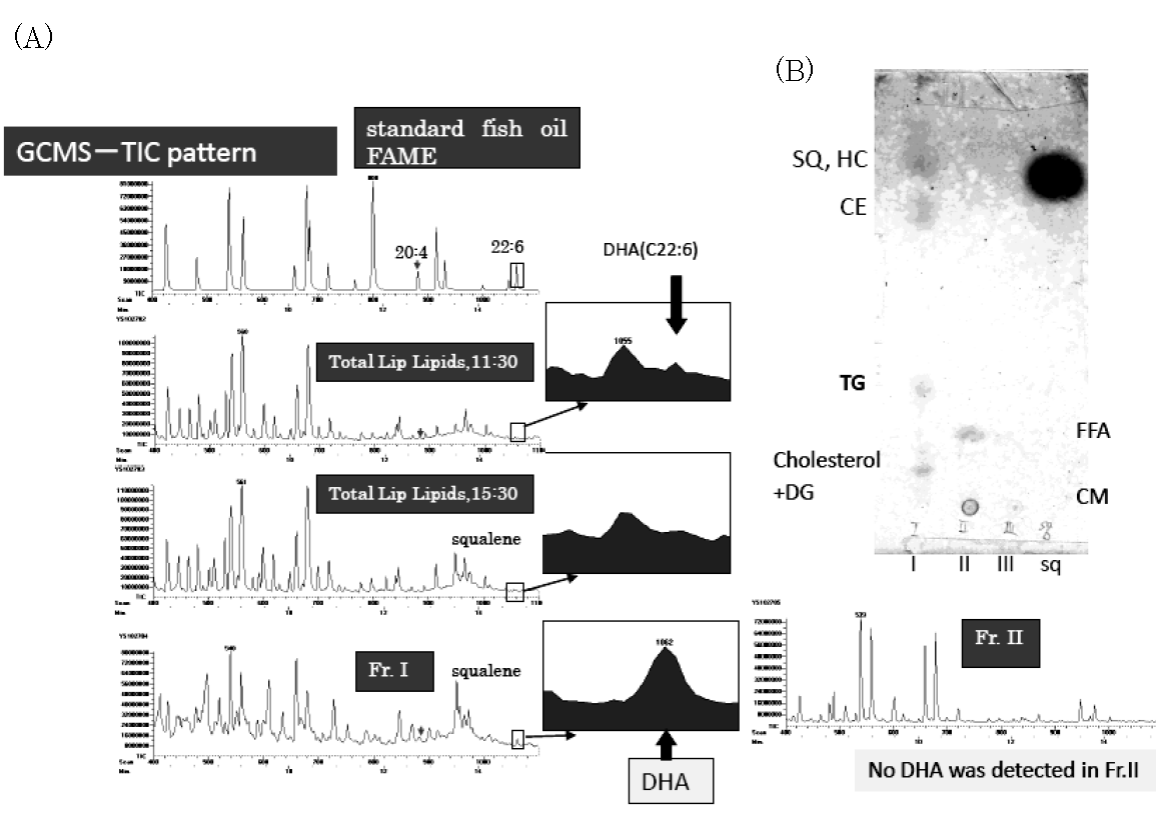
**(A). Fractionation of lip lipids by Silica cartridge and detection of docosahexaenoic acid in Fraction I**. (*left top*) total ion chromatography (TIC) pattern of standard fish oil fatty acid methyl esters. (*left second top*) TIC of total fatty acid methyl esters from total lipid extract of lip surface obtained at 11:30 clock time. (*left third top*) TIC of total fatty acid methyl esters from lipid extract obtained at 15:30 clock time. (*left bottom*) TIC of Fraction I fatty acid methyl esters. The peak of DHA methyl ester was clearly observed as shown in the inserted figure (enlarged chromatogram). (*right bottom*) TIC of Fraction II fatty acid methyl esters and almost no DHA peak was observed. **Fig. 3(B)**(*right top*) TLC patterns of lipids isolated in Fraction I, II, and III by silica gel cartridge. Squalene was applied in the sq lane. Developmental solvent was the same as Fig. 2.

Fig. 2 shows typical TLC pattern of lip lipids extracted with EPI solvent, and the lip lipids contained mainly hydrocarbons, squalene, cholesterol ester, triglycerides, free fatty acids, free cholesterol, diglycerides, and polar lipids. In this TLC pattern of lip lipids, hydrocarbons and squalene were developed in the nearly same portion as cholesterol ester and not well separated. However, the presence of hydrocarbons and squalene in the lip lipids was detected by gas chromatography (as shown in Fig. 3 later). As shown later, this lipid fraction contained lipid hydroperoxides significantly and a faint band above free cholesterol band may correspond to the lipid hydroperoxide. The polar lipids of lip were mainly ceramides (data not shown) and phospholipids could not be detected.

### Measurement of DHA metabolism in situ

It was necessary to know the fate of dietary docosahexaenoic acid (DHA) and we determined which lipid fractions of the lip contained DHA. The amount of DHA of the lip changed depending on duration time from the time of intake of the diet containing 600 mg DHA and 150 mg eicosapentaenoic acid (EPA).

Fig. 3(A) shows gas chromatographic patterns of fatty acid methyl esters derived from fractionated lipids of lip by silica cartridge. The inserted TLC pattern (Fig. 3(B)) shows the lipid profiles of fraction I, II, and III isolated from silica cartridge, and the standard squalene. Here free fatty acids and relatively polar lipids such as ceramides were observed in fraction II or III. Other neutral lipids (squalene, hydrocarbon, cholesterol ester, triglyceride, free cholesterol and diglyceride) were observed in the fraction I. The gas chromatographic patterns of total lip fatty acids collected at 11:30 and 15:30 clock time were shown in the middle two patterns. The peak of docosahexaenoic acid methyl ester (DHA) was larger in the sample obtained at 11:30 clock time than that at 15:30 clock time. The peak of DHA was clearly observed in the fraction I lipids, but not in the fraction II lipids. This suggests that dietary DHA fatty acid was not observed in the free fatty acid fraction of lip surface lipids during metabolic process within 4 to 6 hours from the uptake of DHA in triglyceride form.

**Figure 4:**
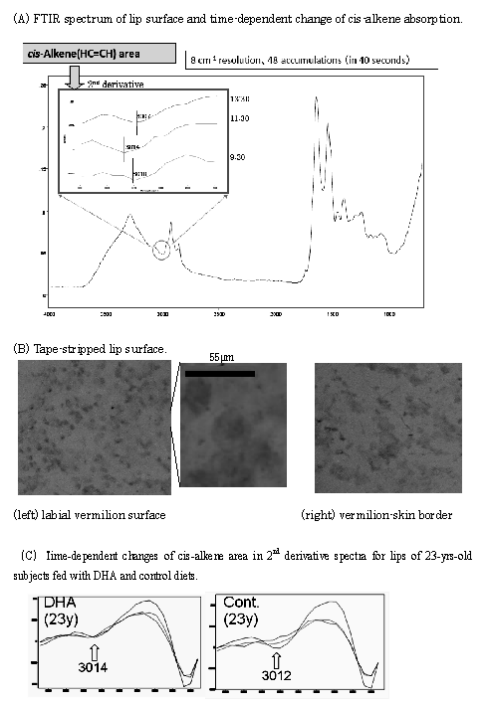
**FTIR spectrum of lip surface and detection of polyunsaturated fatty acids and surface ultrastructures**. **(A) **FTIR spectrum of lip surface; The inserted figure shows the second derivative spectra for cis-alkene absorption area measured at 9:30, 11:30, and 13:30 clock time. FTIR spectra was measured in 8 cm^-1 ^resolution and 48 accumulations within 40 seconds. **(B) **Tape-stripped lip surfaces stained by Alcian blue. (*left*) labial vermilion surface: (*middle*)enlarged spot of a part of left figure: (*right*) labial vermilion-skin border surface. The left and right pictures were taken by stereomicroscope (low mag), and the middle was by inverted microscope (Olympus, Japan) with hemocytometer cover slip (Cellocate, Eppendorf) as a field width indicator. The spot size was 20~40 μm in diameter. **(C) **2^nd ^derivative spectra in cis-alkene area for lips of 23-yrs old subjects with DHA and control diets. Docosahexaenoic acid shows typical cis-alkene IR absorption at 3014 cm^-1^, and arachidonic acid shows at 3012 cm^-1^.

Fig. 4(A) shows a typical FTIR spectrum of lip surface measured *in situ*. The inserted figure shows the second derivative FTIR spectra at around 3010 cm^-1 ^of lip surface at 9:30, 11:30, and 13:30 clock times. The IR absorptions at around 3010 cm^-1 ^were assigned to *cis*-alkene (HC = CH) CH-stretching mode [5], and the shift of the *cis*-alkene peak from 3010 cm^-1 ^to 3014 cm^-1 ^and to 3007 cm^-1 ^suggests changes of polyunsaturated fatty acid species [5] on the lip surface in the time-dependent manner, especially the appearance of 3014 cm^-1 ^peak indicates the enrichment of docosahexaenoic acid (DHA) in the lip surface lipids. Fig. 4(B) shows the Alcian blue-stained (sulfated glycosaminoglycans were detected) lip surfaces (vermilion portion and the lip-skin border portion) which were adhesive tape-stripped. In the vermilion portion clear round spot-like staining was observed with 20 ~30 μm in diameter, and this suggested that the labial vermilion surface contained horny layers of keratinocytes. On the other hand, the skin-border portion contained flattened, squamous cell-like layers. Fig. 4(C) shows typical 2^nd ^derivative spectral changes in the *cis*-alkene region for lips of 23 years-old male subjects fed with DHA- and control-yogurt diets. In the DHA diet clear band at 3014 cm^-1 ^was observed, while in the control diet (soy oil diet) the band at 3012 cm^-1 ^was remarkable.

**Figure 5:**
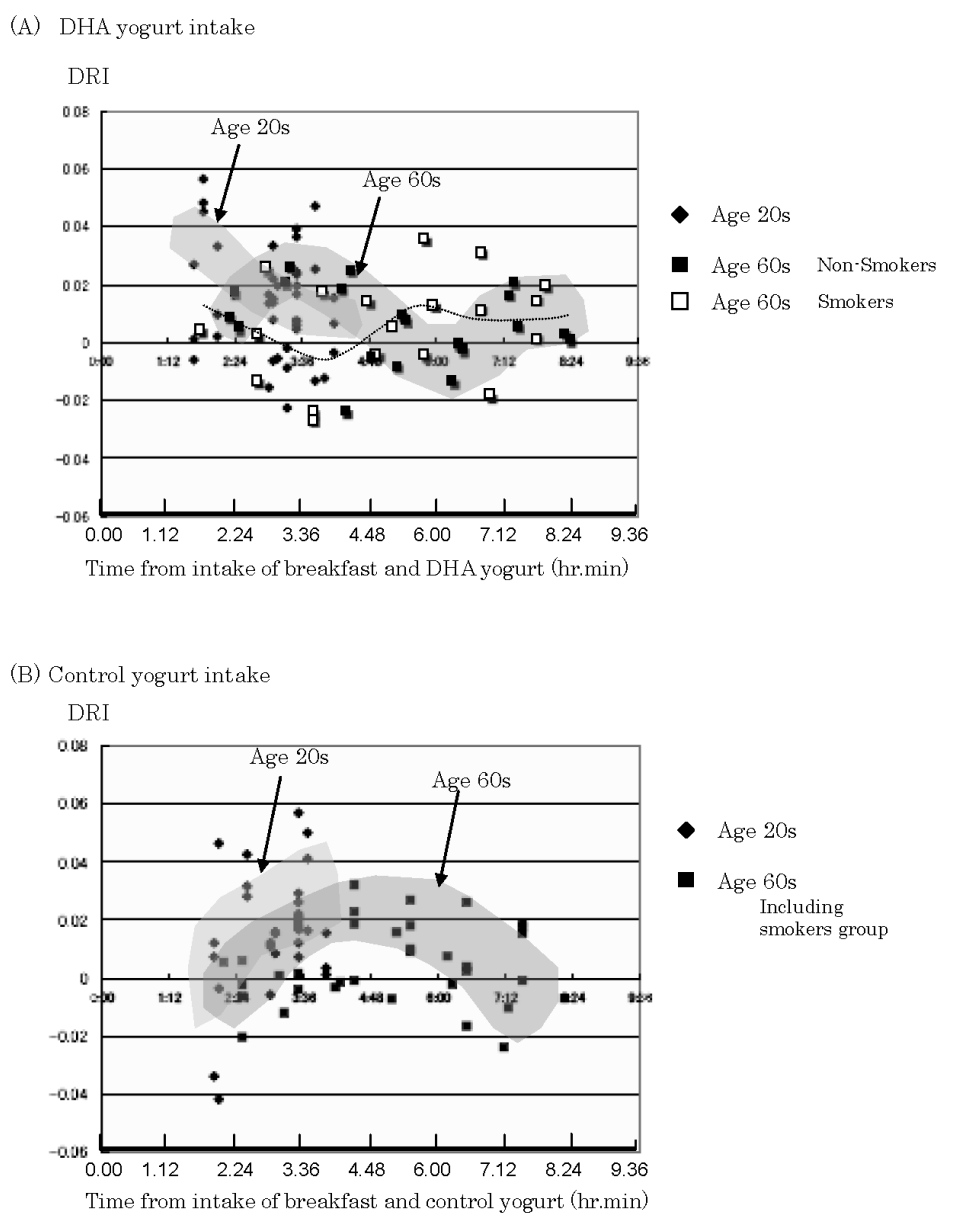
**Time-dependent changes of DRI values for young (age 20s) and old (age 60s) volunteers with DHA-yogurt and control-diets**. **(A) **Duration time (after the intake of breakfast) -dependent patterns for DHA-yogurt diet. The data of DRI changes for subjects with age 20s (12 male persons, all were non-smoking; the duration times were within 4 hours.) and age 60s (6 male persons; three in non-smoking group and the other three in cigarette smoking group) were represented, and the start points for age 20s group were higher than age 60s. **(B) **DRI changes for the control diet. DRI values for age 20s group were also higher than for age 60s in 2~4 hours after the breakfast, and the maximum level of DRI was obtained in 4~6 hours for age 60s, suggesting the increase of arachidonic acid.



Fig. 5 shows changes of DRI values for age 20s (n = 12 × 3; twelve persons and three measurements) and 60s (n = 6 × 6; six persons and six measurements) with DHA diet and control (soy oil) diet. DRI (DHA-Relative Intensity) value was calculated using the following formula:

Here, [3010], [3022], [3014], [2979], and [2960] indicate the intensities at 3010, 3022, 3014, 2979, and 2960 cm^-1^, respectively, in the second derivative spectrum, and actually this DRI value corresponds to the relative intensity of DHA-derived *cis*-alkene peak against methyl CH antisymmetric stretching mode peak at 2960 cm^-1 ^originated mainly from tissue surface proteins. For DHA diet, large DRI values were detected for age 20s only at 1.5 ~2 hours of duration after intake of breakfast with DHA yogurt, whereas the increase of DRI was detected for age 60s group (cigarette non-smoking group; n = 3) at 3 ~4 hours of duration after intake of breakfast with DHA. However, the increase of DRI was detected only at 5 ~6 hours of duration for age 60s group (cigarette smoking group; n = 3) after intake of breakfast with DHA yogurt.

On the other hand, the increase of DRI values was detected at 3 to 4 hours of duration for both age 20s and 60s groups in the control diet using soy oil (0.6 g)-containing yogurt. In this case of control diet, the increase of arachidonic acid (C20:4) may affect the FTIR spectrum at around 3010 cm^-1 ^and may contribute to the increase of DRI values. Smoking or non-smoking did not affect significantly the change of DRI values for age 60s group in the control diet.

These results suggest that dietary DHA metabolism may be detected non-invasively with measuring *cis*-alkene FTIR spectra of lip surface of human.

### Measurement of lipid hydroperoxides on lip surface

Before measurement of lipid hydroperoxides (LOOH) by FTIR *in situ*, we tried to detect lipid hydroperoxides extracted from lip surface biochemically.

**Figure 6:**
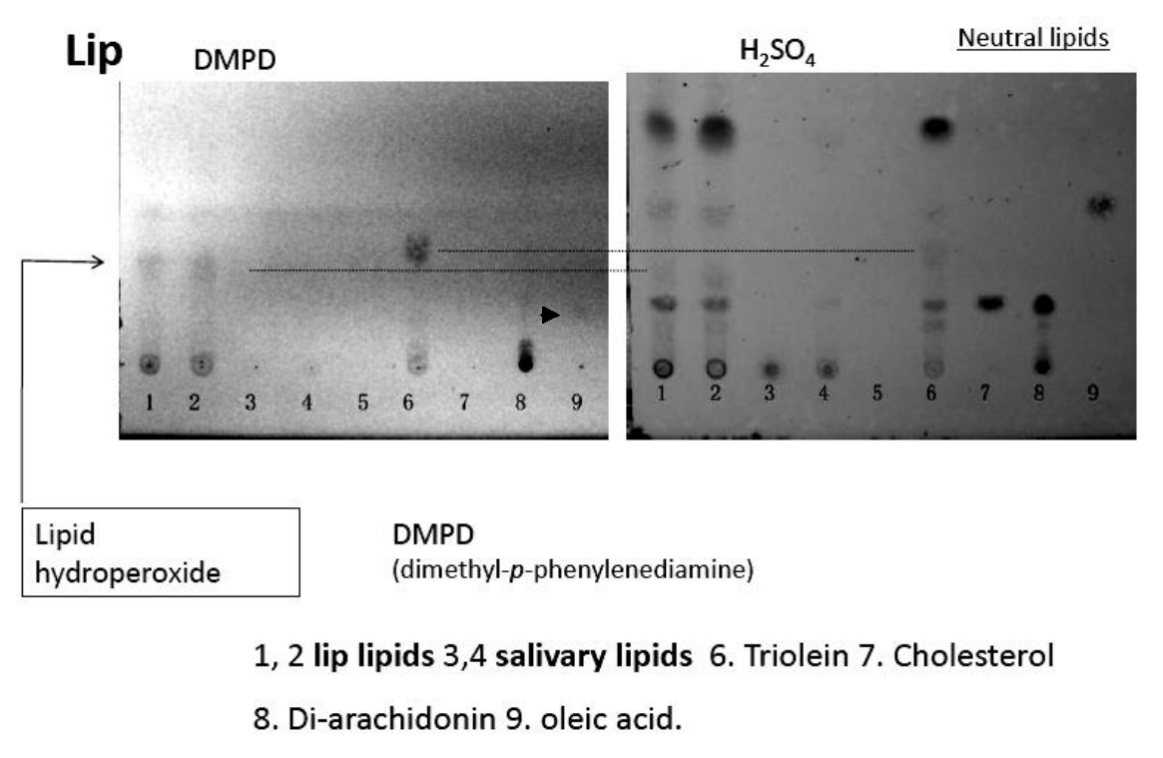
**Detection of lipid hydroperoxides in lip by DMPD staining**. Originally DMPD (N, N,'-dimethyl-*p*-phenylenediammonium dichloride) stains lipid hydroperoxides in reddish pink, and partly oxidized tri-olein (TG-hydroperoxide) standard in lane 6 was observed by DMPD staining just above the position of free cholesterol or diglyceride. The spot of lipid hydroperoxide in lip lipids was observed near the TG-hydroperoxide spot. The arrow head shown in the left figure suggest the presence of oleic acid hydroperoxide. The salivary lipid extract did not show hydroperoxide spot under this developmental condition.

Fig. 6 shows TLC patterns of DMPD-stained lipid hydroperoxides (left) and of charred lipids by sulfuric acid (right) for mainly neutral lipids from lip surface, saliva, and standards. Originally DMPD-stained lipid hydroperoxide showed pink colored spots and here presented in black-white photo. Among standard lipids, a little aged triolein and diarachidonin contained hydroperoxides and these were clearly detected by DMPD staining as shown Fig. 6 (left, lane 6 and 8). The hydroperoxide of free oleic acid was faintly detected as shown by an arrow head. Hydroperoxide spot in triglyceride lane was mainly observed just above the cholesterol spot, and that in diarachidonin was observed at the origin (relatively polar lipid fraction) and near monoglyceride position. Those spots may correspond to triglyceride hydroperoxide in lane 6 and diglyceride hydroperoxide in lane 8. Lipid hydroperoxides of lip surface lipids were observed in lane 1 and 2 just near the triglyceride hydroperoxide. In salivary lipids, no spots of lipid hydroperoxides were observed in neutral lipid area, but a faint spot was observed at the origin. Skin lipids extracted from face forehead, not shown here, did not show spot at triglyceride hydroperoxide, but at more upper part, possibly squalene hydroperoxide [6,7] very faintly.

Next the lipid hydroperoxide near the spot of triglyceride hydroperoxide in the lip surface lipids was quantitated after charring through measuring spot intensities and compared with other neutral lipids.

**Figure 7:**
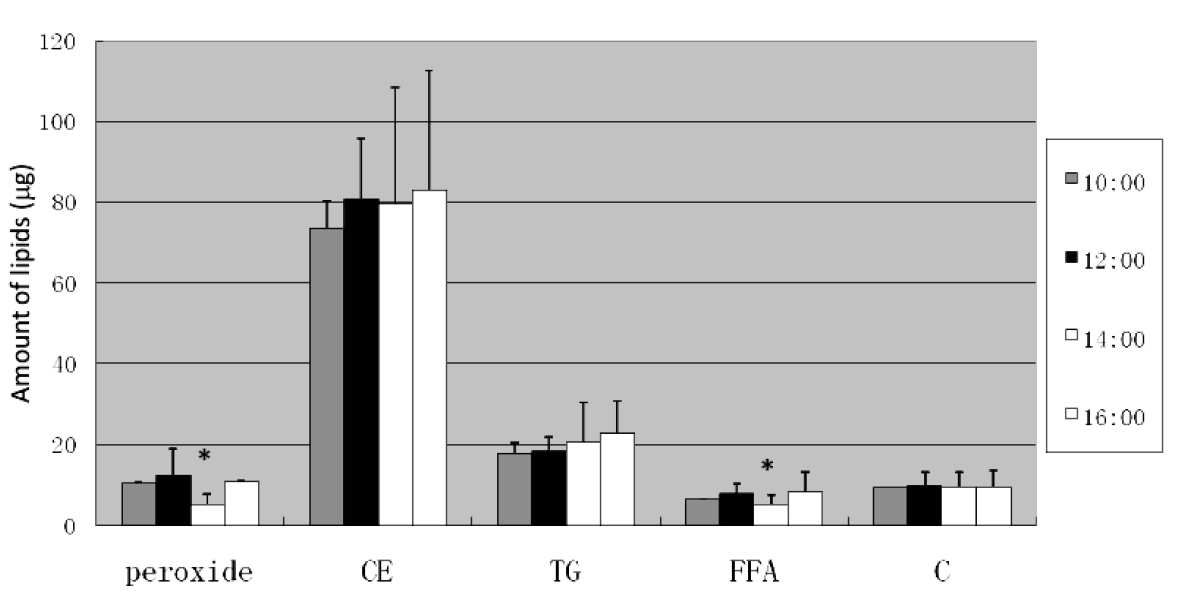
**Clock time-dependent changes of neutral lipids (cholesterol ester, CE; triglyceride, TG; free fatty acid, FFA; free cholesterol, C) and lipid hydroperoxide of lip surface**. Certain amount of internal standard elaidic acid methyl ester, which appears between the spots of triglyceride and cholesterol ester in the TLC, was mixed in the PVDF membrane after extraction of lip lipids and co-extracted as shown in Fig. 1. These neutral lipids were developed on TLC with hexane:diethylether:acetic acid (80:30:1) and charred by 50% sulfuric acid at 170°C for nearly 30 min. The TLC patterns were incorporated in computer with scanner, and the charred spots were quantitated in intensity with Scion Image software (Scion Co., Israel) using a gel-plotting macro program. The amounts of the neutral lipids in μg unit were calculated in comparison with the amount of the internal standard, 20 μg of elaidic acid methyl ester, and under the present condition the extent of darkness of charred spot (after conversion to optical density by Scion Image program) was linearly well correlated with amount of neutral lipids. In this case the amount of lipids in lip thus calculated was correspond to the amount of lipids extracted to the PVDF membrane in one wiping event of the lip surface.

Fig. 7 shows the clock-time dependent changes of lipid hydroperoxide and neutral lipids and this suggested that lipid hydroperoxide and free fatty acids were significantly decreased at 14:00 clock time and other lipids were nearly constant. Here, data were analyzed statistically using Student's t-test (n = 6) for the data at 12:00 and 14:00 clock times.

**Figure 8:**
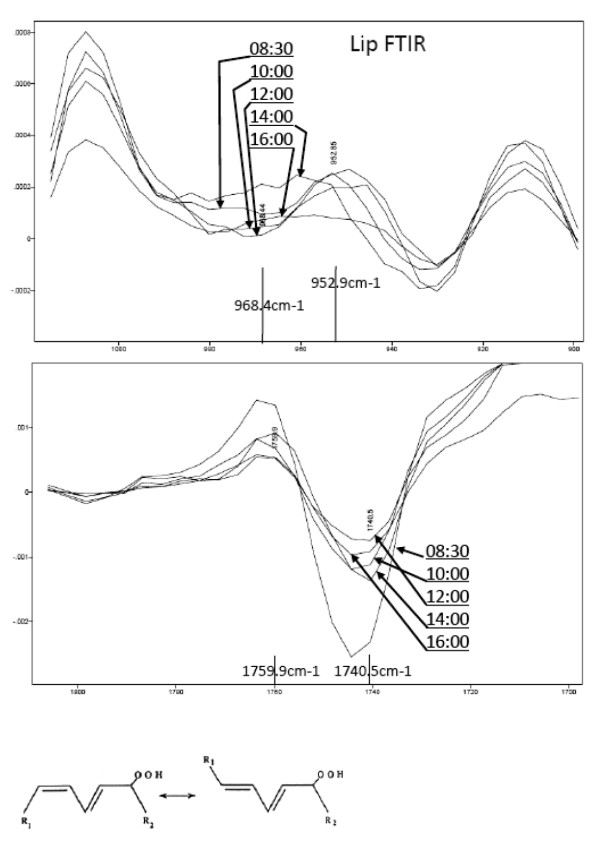
**FTIR spectra in the second derivative form of lip surfaces measured at different clock times**. (*top*) Spectral changes in the area around 960 cm^-1^, related to trans-alkene CH-stretching mode. The difference between the intensities at 952.9 and 968.4 cm^-1 ^was calculated and denoted as *trans*-alkene intensity or [hydroperoxide]. (*middle*) spectral changes in the area around 1740 cm^-1^, related to fatty ester mode. The difference between the intensities at 1759.9 and 1740.5 cm^-1 ^was calculated and denoted as fatty ester intensity or [TG]. (*bottom*) illustration of lipid hydroperoxide structure, indicating the presence of *trans*-alkene in the structure.

**Figure 9:**
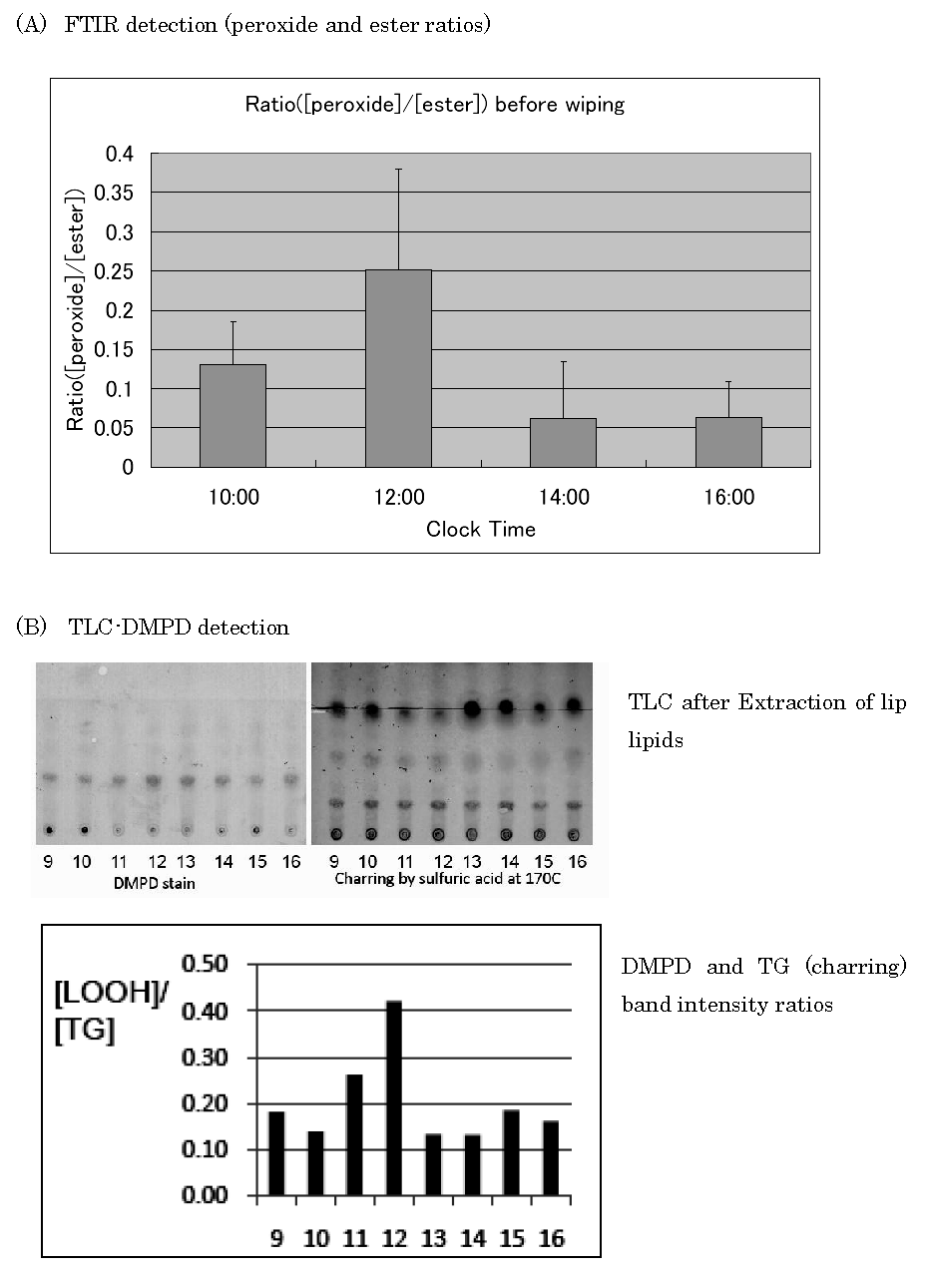
**Comparison between FTIR and TLC detection methods of clock-time dependent changes of lipid hydroperoxides in the lip**. **(A) **Clock time dependent changes of infrared spectral ratio between the peroxide-related *trans*-alkene absorption at 968 cm^-1^, [peroxide], and the triglyceride-related ester absorption at 1740 cm^-1^, [ester]. The ratio was the highest at noon just before lunch. The subjects were two males in age 20s and 30s and the increase of the ratio at 12:00 clock time was significant (n = 4, p < 0.05) when compared with the ratio at 10:00 or 14:00 clock time. **(B) **Clock time dependent TLC patterns (*right inserted figure*; DMPD stained LOOH (*left side*) and charred TG (*right side*)) and [LOOH]/[TG] ratio changes (*left inserted figure*). The lip surface lipids were extracted as shown in Fig. 1(B) and developmental condition for TLC was the same as shown in Fig. 2. The subject was one male in age 30s. Both the clock time dependent patterns in FTIR and TLC were quite resembled, however, the experiment of (B) was done only in duplicate and the statistical consideration was avoided.

Next we tried to measure FTIR spectra of lip surface by CISME measurement system to detect the change of lipid hydroperoxides *in situ*. Fig. 8 shows an example of time-dependent changes of FTIR spectra in the second derivative forms at around 960 cm^-1 ^(top figure) and at around 1740 cm^-1 ^(bottom figure). It is well known that *trans*-alkene (HC = CH) in fatty acids of foods shows infrared absorption at 966~968 cm^-1 ^[8,9], whereas the *cis*-alkene shows at around 3010 cm^-1 ^as shown in the above section. Normally the formation of lipid hydroperoxide accompanies the occurrence of *trans *conjugation of double bond as shown in the bottom inserted figure. The upper figure of Fig. 8 showed the clock-time-dependent changes of FTIR spectra (second derivative form) at around 960 cm^-1^, suggesting the increase (to downward) of 968 cm^-1 ^band at 12:00 clock time. Actually the difference value in the second derivative spectra was calculated between the intensities at 952.9 and 968.4 cm^-1^, and this difference value was divided by the intensity at 1740 cm^-1 ^(the difference between intensity values at 1759.9 and 1740.5 cm^-1^), originated from fatty ester form mainly. This value may correspond to the ratio between lipid hydroperoxide and esters, and summarized in Fig. 9.

Fig. 9(A) shows the clock-time dependent changes of the peroxide to ester ratios measured by FTIR *in situ*, and Fig. 9(B) shows also the clock-time dependent changes of biochemically detected peroxides and the ratio between lipid hydroperoxides (LOOH) and triglyceride (TG) bands in TLC. The lipids of lip surface were extracted as described above (shown in Fig. 1(B)) and the lipid hydroperoxides were detected by DMPD staining and total lipids were stained by sulfuric acid. Those stained intensities were compared and the band intensity ratio ([LOOH]/[TG]) between LOOH and TG was calculated. The extracted lipid samples were collected at different clock times in different days; at 1^st ^day, lipids were collected at 9:00, 12:00, 14:00, 16:00, and at 2^nd ^day at 11:00, 13:00, 15:00, and 3^rd ^day at 10:00, under quite similar dietary conditions (menu, the amount, and clock time of food intakes) in those days. This result indicated that [LOOH]/[TG] ratio was the highest at 12:00 clock time, just before lunch, and in this case the amount of triglyceride was decreased at 12:00. This biochemical profile of the time course was consistent with the spectral profile obtained from peak ratio in the FTIR spectra, where the ratio between *trans*-alkene peak at 968 cm^-1 ^and the ester peak at 1740 cm^-1 ^was the highest at 12:00 clock time.

These results indicate that the measurement of lip surface FTIR *in situ *could detect the change of lipid hydroperoxide on lip surface.

## Discussion

The present paper has described that the measurement of FTIR spectra of human lip surface could detect the metabolism of dietary fatty acids, especially docosahexaenoic acid (DHA), and the change of lipid hydroperoxides *in situ *non-invasively. Normally the metabolism of dietary fatty acids has been measured so far using human blood cells and stable-isotope labeled fatty acids [10], and it was shown that dietary DHA in triglyceride was incorporated into very-low-density-lipoprotein (VLDL)-triglyceride within 2 hours, and very slowly (more than 60 hours) into red blood cell phosphatidylcholine [11]. Moreover, dietary DHA in phosphatidylcholine was incorporated maximally into triglyceride of human plasma in 6 hours after digestion and negligible incorporation into cholesterol ester was observed. These data for human body were only obtained using blood (plasma or cells), and so far no data available for human peripheral tissues and other major organs.

We found as shown in this paper that the metabolism of dietary DHA or other fatty acids/lipid hydroperoxides could be measured non-invasively through human lip. As lip surface contains thinner stratum corneum (SC) in the vermilion zone (red zone) with less barrier functions [12,13] than normal skin SC and the red color of lip is unique to human and comes from the blood vessels in the dermis, it may be expected that the penetration or transport of biomaterials (water, electrolytes, sugars, lipids and others) from blood vessels or reuptake from the lip surface to blood vessels is more rapid than other body skin. There is a report for the permeability of solutes to human skin layers [14-16]which showed that the permeability coefficient increased with increasing the lipophilicity.

However, we have no data available so far to estimate the penetration and diffusion coefficient of chemicals *in situ *for human lips. Generally human lip has 4 portions anatomically, (1)appendage-bearing epidermis, (2)keratinized vermilion (red zone), (3)parakeratinized intermediate zone, and (4)labial mucosal epithelium [17]. In the present paper, we measured the lower lip, keratinized vermilion surface (outermost stratified corneum) by FTIR-ATR method, and this portion was suitable to measure the metabolism of polyunsaturated fatty acids originated from diet. This vermilion portion normally contains several layers of partly keratinized epithelia with horny, villous appearance [17], and partly transparent to see the red color of lip vessels.

As shown in this paper, the dietary fatty acids, such as DHA, could be transported from blood vessels to the lip (labial vermilion) surface. However, the appearance of dietary DHA on the face skin surface could not be detected by the same FTIR-ATR method (data not shown). Normal skin surface fatty acids may be originated mainly from sebum. Because it would take time to incorporate dietary lipids from blood and synthesize lipids in sebaceous glands of skin, it may be hard to detect the dietary DHA or other polyunsaturated fatty acids of blood on the skin surface. On the other hand, the lip vermilion surface lipids may not be from sebum, because sebaceous and sweat glands in the vermilion portion were rare histologically.

The transport or movement of dietary DHA from blood vessels to the lip surface was unexpectedly rapid for younger men as shown in Fig. 5 and within 2 hours we could detect the dietary DHA on the lip surface after the intake of DHA containing diet. On the other hand, it took 4 or 5 hours for older men to detect dietary DHA on the lip surface, and this difference of transport speed of DHA between younger and older men may not be explained simply by the structural difference of lip stratum corneum, such as the intercellular matrix or lamellar membranes. As shown in Fig. 5, the time-dependent DRI changes in the control diet were similar in both, younger and older, ages groups, and the increase of polyunsaturated fatty acids of lip surface, mainly arachidonic acid (C20:4) and linoleic acid (C18:2), could be detected at 3 to 5 hours after the intake of control diets.

This difference in transport rate between DHA and other fatty acids from blood vessels to lip surface may not be simply explained by a passive penetration mechanism of DHA, but we may have to assume the presence of specific transport mechanism of DHA. The transport of DHA from blood vessels to neonate is reported to be dependent on the expression of fatty acid-transport proteins in placenta [18] and that lipid carriers were involved in placental transfer of DHA. It was also reported that the requirement of DHA for brain development and the coincident expression of brain lipid-binding protein (BLBP) during developmental stages indicated the involvement of BLBP in the utilization of DHA in brain [19].

Although the role of such fatty acid-binding protein is not known in transport of lipids from blood vessels to lip, a facilitated transport mechanism of DHA in human lip may be present as shown in this paper and this transport mechanism may be different from that of other fatty acids, such as arachidonic acid. We demonstrated also that the transport of DHA to the lip vermilion surface was carried out mainly in the form of triglyceride (or less in diglyceride form). More detailed analyses may be needed using our newly developed FTIR-ATR apparatus.

Not only polyunsaturated fatty acids were transported to lip surface but also a significant amount of LOOH was detected on the surface. These lipid hydroperoxides were non-destructively measured *in situ *by FTIR-ATR, and the LOOH/TG ratios were increased just before noon in our experiments. The lip lipid hydroperoxides were probably triglyceride form mainly as shown in Fig. 9. We did not determine whether the lipid hydroperoxides were dietary-origin, or metabolized and circulating lipids-origin in a body, and whether the lipid hydroperoxides were formed just on the lip surface by exposure to oxygen or UV light or other environmental pollutants (*ex*., cigarette smoke). In any case we could not assume the lip lipid hydroperoxides were solely dietary origin, because the increase of lipid hydroperoxides was not observed in the afternoon even at 4 hours later after lunch. In both breakfast and lunch, these diets normally contained significant amount of linoleic acid (C18:2) even in bread or rice, a base diet. The drastic increase of LOOH/TG ratio just before noon on the lip may thus indicate a physiological phenomenon. As PUFA in triglyceride of lip surface may have a rhythm to be increased just before noon or 4 to 5 hours after breakfast under the normal diet as shown in Fig. 5 in this paper, this increase of PUFA on lip surface may be a part of causal phenomena for the increase of lipid hydroperoxides. However, the precise mechanism for appearance of lipid hydroperoxides on lip vermilion surface is not clear at present.

It has been known that lipid hydroperoxides on the normal skin like face forehead is derived from mainly cholesterol [20] and squalene [6,7], and the skin squalene hydroperoxide was enhanced by illumination of ultraviolet light. Normally skin squalene hydroperoxide produced could be only detected by high sensitive methods such as chemiluminescence [20,21] and mass spectrometric [7] methods. However, the amount of skin squalene hydroperoxide was very little and we could not detect it by the present DMPD staining method on TLC (data not shown) under the condition where lip lipid hydroperoxide was detected with extracting lipids using PVDF membrane and EPI solvents.

## Conclusion

We have demonstrated that our newly developed FTIR-ATR system could detect non-invasively and easily the metabolic changes of polyunsaturated fatty acids and lipid hydroperoxides in human body through lip vermilion surface. It was observed that dietary DHA in triglyceride was transported to the lip surface more rapidly for men with age 20s than age 60s, and that lipid hydroperoxides of lip vermilion were increased just before noon with correlation to the increase of polyunsaturated fatty acids.

## Abbreviations

DHA: docosahexaenoic acid; DMPD: N, N'-dimethyl-*p*-phenylenediammonium dichloride; DRI: DHA-relative intensity; EPA: eicosapentaenoic acid; EPI: the solvent mixture of ethanol/ethylpropionate/iso-octane; FTIR-ATR: Fourier transform infrared spectroscopy with attenuated total reflectance; LN: linoleic acid; LOOH: lipid hydroperoxide; PVDF: polyvinylidenedifluoride; SC: stratum corneum; TG: triacylglycerol or triglyceride.

## Competing interests

The authors declare that they have no competing interests.

## Authors' contributions

SY has made substantial contribution to conception, design, acquisition of data, analysis and interpretation of data, drafting the manuscript, and final approval of the version to be published. QZZ carried out TLC and GCMS analyses for fatty acids and lipids in lips. SS participated in the design of the skin and lip studies. SM carried out the TLC, GCMS, and FTIR measurements for lip lipids and fatty acids. All authors read and approved the final manuscript.
